# Circular RNA hsa_circ_0044234 as distinct molecular signature of triple negative breast cancer: a potential regulator of GATA3

**DOI:** 10.1186/s12935-021-02015-6

**Published:** 2021-06-14

**Authors:** Farzaneh Darbeheshti, Elham Zokaei, Yaser Mansoori, Sima Emadi Allahyari, Zeeba Kamaliyan, Sepideh Kadkhoda, Javad Tavakkoly Bazzaz, Nima Rezaei, Abbas Shakoori

**Affiliations:** 1 Department of Medical Genetics, School of Medicine, Tehran University of Medical Sciences, Tehran, Iran; 2 Medical Genetics Network (MeGeNe), Universal Scientific Education and Research Network (USERN), Tehran, Iran; 3 Department of Biology, Faculty of Sciences, Shahid Bahonar University of Kerman, Kerman, Iran; 4 Noncommunicable Disease Research Center, Fasa University of Medical Sciences, Fasa, Iran; 5 Department of Medical Genetics, Fasa University of Medical Sciences, Fasa, Iran; 6 Department of Medical Genetics, School of Medicine, Shahid Beheshti University of Medical Sciences, Tehran, Iran; 7 Research Center for Immunodeficiencies, Children’s Medical Center, Tehran University of Medical Sciences, Dr. Qarib St, Keshavarz Blvd, Tehran, Iran; 8 Department of Immunology, School of Medicine, Tehran University of Medical Sciences, Tehran, Iran; 9 Network of Immunity in Infection, Malignancy and Autoimmunity (NIIMA), Universal Scientific Education and Research Network (USERN), Tehran, Iran; 10 Medical Genetic Ward, Imam Khomeini Hospital Complex, Tehran University of Medical Sciences, Dr. Qarib St, Keshavarz Blvd, Tehran, Iran; 11 Breast Disease Research Center (BDRC), Tehran University of Medical Sciences, Tehran, Iran

**Keywords:** Triple negative breast cancer, circ_0044234, miR135b-5p, GATA3, ceRNA, Bioinformatics

## Abstract

**Background:**

Circular RNAs (circRNAs) have been implicated in the initiation and development of breast cancer as functional non-coding RNAs (ncRNA). The roles of circRNAs as the competing endogenous RNAs (ceRNAs) to sponge microRNAs (miRNAs) have also been indicated. However, the functions of circRNAs in breast cancer have not been totally elucidated. This study aimed to explore the clinical implications and possible roles of circ_0044234 in carcinogenesis of the most problematic BC subtype, triple negative breast cancer (TNBC), which are in desperate need of biomarkers and targeted therapies.

**Methods:**

The importance of circ_0044234 as one of the most dysregulated circRNAs in TNBC was discovered through microarray expression profile analysis. Reverse transcription-quantitative polymerase chain reaction (RT-qPCR) was performed to confirm the downregulation of circ_0044234 in triple negative tumors and cell lines versus non-triple negative ones. The bioinformatics prediction revealed that circ_0044234 could act as an upstream sponge in the miR-135b/GATA3 axis, two of the most dysregulated transcripts in TNBC.

**Results:**

Our experimental investigation of circ_0044234 expressions in various BC subtypes as well as cell lines reveals that TNBC expresses circ_0044234 at a substantially lower level than non-TNBC. The ROC curve analysis indicates that it could be applied as a discriminative biomarker to identify TNBC from other BC subtypes. Moreover, circ_0044234 expression could be an independent prognostic biomarker in BC. Interestingly, a substantial inverse expression correlation was detected between circ_0044234 and miR-135b-5p as well as between miR-135b-5p and GATA3 in breast tumors.

**Conclusions:**

The possible clinical usefulness of circ_0044234 as a promising distinct biomarker and upcoming therapeutic target for TNBC have been indicated in this research. Our comprehensive approach revealed the potential circ_0044234/miR135b-5p/GATA3 ceRNA axis in TNBC.

**Supplementary Information:**

The online version contains supplementary material available at 10.1186/s12935-021-02015-6.

## Background

Breast cancer (BC) is one of the most common cancers in women and the second leading cause of cancer-related deaths worldwide [1]. Human breast cancers demonstrate a heterogeneous group of tumors with varying behaviors, symptoms, and therapeutic responses. Based on the genes expressed, there are four major intrinsic or molecular subsets of breast cancer, including luminal A/B, HER2-enriched**,** and triple negative (TN) [2]. Triple negative breast cancer (TNBC) is the poorest prognostic subtype of breast cancer, accounting for about 15% of all cases. It is distinguished by the lack of human epidermal growth factor receptor 2 (HER2), progesterone receptor (PR), and estrogen receptor (ER) expressions [3, 4]. Regarding disease management in these patients, the medical advances have lagged other molecular subtypes. It is related to a high risk of invasion, early disease recurrence, and short survival in TNBC patients [5, 6]. Early detection of cancer by successful diagnostic markers improves the survival rate of breast cancer patients. However, the field of biomarker research for early detection, prognosis, and prediction of treatment response in TNBC is rapidly expanding. Efficient biomarkers for routine clinical management of TNBC have not yet been established [7, 8]. Our knowledge about the transcriptomic profiles of tumor cells involved in tumorigenesis can help us develop more effective biomarkers and treatments.

Covalently closed circular RNAs (circRNAs) are a novel competing endogenous noncoding RNA (ncRNAs) [9]. They have been implicated in a class of RNAs with tissue/developmental stage-specificity, making circRNA a potential novel biomarker for cancer diagnosis and prognosis. Due to no free 5′ and 3′ ends in their structure, circRNAs show more stability than traditional linear RNAs when exposed to RNase, facilitating circRNAs to exert their regulatory function at the transcriptional and post-transcriptional levels [10]. Thanks to technological breakthroughs in high-throughput sequencing and computational approaches, circRNAs have drawn increasing interest, revealing the existence of a large amount of previously unidentified circRNAs. Exonic circRNAs exist abundantly in the cytoplasm and are considered microRNA (miRNA) sponges [11, 12]. Recent evidence has proposed that competitive endogenous RNA (ceRNA) can modulate miRNA activities and regulate gene expression by sequestering specific miRNAs or buffering their repression of mRNA targets. In this dynamic network, ncRNAs with miRNA response elements (MREs) act as sponges for specific miRNAs and restore the expression of their downstream targets [13, 14].

The sequencing data from a large cohort of BC patients reveals specifically expressed circRNAs in different subtypes of BC and the strong connection of tumor proliferation degrees with the expression rate of total circRNAs [15]. In the current study, the microarray dataset from the NCBI Gene Expression Omnibus (GEO) was analyzed in order to identify the difference between circRNA expression profiles in TNBC and non-TNBC. Among differentially expressed circRNAs (DECs), circ_0044234 was picked out for further investigations. Finally, we built an integrative approach to finding the possible ceRNA axis for circ_0044234 in TNBC based on our previous findings and evaluated their expression signature in the different subtypes of BC [16].

## Methods

### Evaluation of circular RNA expression profiling in triple negative breast cancer through microarray data analysis

Firstly, using the GEO database, the raw data from the microarray dataset GSE101124 was obtained, which included the expression profile of circRNA in triple negative and luminal breast tumor samples. Subsequently, the Limma package in R software was used to analyze differentially expressed circRNAs in TNBC relative to luminal tumors to assess discriminative DECs in TNBC. The DECs were classified as circRNAs with a log_2_^fold change^ more than |1| and a *P*-value less than 0.05.

### Patient samples

The ninety-five fresh frozen samples of breast tumors were obtained from Cancer Institute, Imam Khomeini Medical Center, Tehran, Iran. The collected samples include 40 triple negative, 20 luminal A, 18 luminal B, and 17 HER2^+^ subtypes. Prior to surgery, none of the patients had undergone any hormone therapy or chemotherapy. All the collected tissues have been pathologically approved by histopathological analysis. Additionally, ten “true normal” samples were obtained from healthy women who had undergone cosmetic mammoplasty. This study was approved by the Ethics committee of Tehran University of Medical Sciences (TUMS) (Code of Ethics: IR.TUMS.MEDICINE.REC.1398.792) and written consent was obtained from all patients. Clinical characteristics including age at diagnosis, tumor size, ER/PR/HER2 status, grade, lymph node metastasis (LNM), and histologic type of tumor were attained from hospital records.

### Cell culture

MCF7 (luminal A) and MDA-MB-361 (luminal B) cell lines were cultured in RPMI, while MDA-MB-468 (triple negative) and MDA-MB-231 (triple negative) cell lines were cultured in DMEM-Hi glucose (Cegrogen Biotech, Germany). Then added 10% fetal bovine serum (FBS) and 1% antibiotic/antimycotic solution (Cegrogen Biotech, Germany) to the medium. MCF10-A, a normal breast mammary cell line, was held in DMEM supplemented with 5% FBS, 1% antibiotic/antimycotic solution, insulin 10 g/ml, EGF 20 ng/ml, hydrocortisone 0.5 mg/ml, and cholera toxin 100 ng/ml as a control. At 37 degrees Celsius, the samples were incubated in an incubator with 5% CO2. The cell lines were bought from the Pasteur Institute and National Cell Bank of Iran (NCBI) (Tehran, Iran).

### RNA extraction and RT‑qPCR assay

Initially, TRIzol reagent (Invitrogen, USA) was used to extract complete RNA from breast tissues (tumor and normal) and cell lines as per the manufacturer’s instruction. Gel electrophoresis and spectrophotometry (NanoDrop 2000, Thermo Scientific, USA) were also applied to determine RNA quality and quantity, respectively. The PrimeScriptTM 1st strand cDNA Synthesis Kit then transformed RNA samples into cDNA (TaKaRa Bio, Japan). The relevant stem-loop RT primers have been used to synthesize cDNA for miR135b and RNU44 (Table 1). Finally, Light-Cycler 96 Roche machine was applied to conduct quantitative real-time PCR using RealQ Plus 2 Master Mix Green low ROX (Ampliqon, Denmark). All reactions were carried out in a 10 μl volume and were duplicated. The primer sequences used in real-time PCR have been shown in Table 1. The circ_0044234 back-splice site was confirmed using Sanger sequencing. PUM1 housekeeping gene was used to normalize the expression of circ_0044234 and GATA3, while the housekeeping gene RNU44 was applied to normalize the expression of miR135b-5p. Using the 2^−ΔΔCt^ method, the relative expression of the transcripts was measured. In this study, normal breast mammary tissues and MCF10A cell line were used as calibrator samples in ∆∆Ct = ∆Ct(sample)—∆Ct(calibrator) formula.

**Table 1:** The primer sequences which used in RT-PCR and quantitative real-time PCR

Target Transcript	Primer type	Sequence (5ʹ→3ʹ)
hsa_circ_0044234	Forward	TTGTGTTGATCTCAGCAAGCTT
	Divergent reverse	CTGAGTGTACAATATACATGTCCCA
miR-135b-5p	Stem loop RT	GTCGTATCCAGTGCAGGGTCCGAGGTATTCGCACTGGATACGACTCACAT
	Forward	CTCGTATGGCTTTTCATTCCT
	Universal reverse	GTGCAGGGTCCGAGGT
RNU44	Stem loop RT	GTCGTATCCAGTGCAGGGTCCGAGGTATTCGCACTGGATACGACAGTCAGTT
	Forward	GAATGCTGACTGAACATGAAGGTC
GATA3	Forward	CAAGCTTCACAATATTAACAGACCC
	Reverse	GGGTTAAACGAGCTGTTCTTG
PUM1	Forward	AGTGGGGGACTAGGCGTTAG
	Reverse	GTTTTCATCACTGTCTGCATCC

### Bioinformatics approaches for predicting miRNA response element (MRE) of Circ_0044234

Previously, we applied an integrative and consensus-building approach to analysis of two miRNA microarray datasets (GSE19536 and GSE86948) that indicated seven differentially expressed miRNAs (DE miRNAs) in TNBC relative to non-TNBC [16]. We realized that miR-135b-5p, the most up-expressed miRNA in TNBC, has a potential MRE on circ_0044234 sequence using Cancer-Specific CircRNA (CSCD) [17] and Circular RNA Interactome (CircInteractome) [18] web resources. The miR-135b-5p is thought to be a probable target of the circ_0044234, and their expression correlation was assessed in breast tumor samples.

### Constructing Circ_0044234 /miR-135b-5p/GATA3 ceRNA axis in TNBC

Since we had previously identified GATA3 as a potential hub node in the TNBC oncotranscriptomic network that is potentially located downstream of miR-135b-5p [16], here we have evaluated the expression signature of circ_0044234/miR-135b-5p/GATA3 axis in the breast tumor samples. Furthermore, the heatmap analysis was used to illustrate the expression patterns of the circ_0044234/miR-135b-5p/GATA3 axis in TNBC versus non-TNBC samples.

### Statistical analysis

SPSS Statistics software v.26 and GraphPad Prism v.8 were used for data evaluation and visualization. The expression data was analyzed using the unpaired sample t-test and ANOVA. The linear correlation between elements of the circ_0044234/miR-135b-5p/GATA3 axis was determined using the Pearson correlation coefficient. The effect of upstream ncRNAs on the expression of downstream targets in the circ_0044234/miR-135b-5p/GATA3 ceRNA network was studied using a logistic regression model. GraphPad Prism v.8 was used to develop the receiver operating characteristic (ROC) curve. The Mann–Whitney test or the Kruskal–Wallis test were applied to investigate the relationship between circ_0044234 expressions and clinicopathologic features. Only tumor grade, LNM, Ki67 status, and tumor subtype (TNBC and non-TNBC) variables with significant P.value in the univariate analysis were adjusted in multivariate analysis of variance (MANOVA) test. The association of circ_0044234 expression with disease-free survival (DFS) of patients was analyzed using the Kaplan–Meier method and log-rank test (data for 87 patients were available during a 20-month follow-up). For the two latter analyses, circ_0044234 logFC was categorized into high (logFC > 1) and low (logFC ≤ 1) groups. Statistical significance was described as a P-value of less than 0.05.

## Results

### Screening of DECs in triple negative breast cancer (TNBC) versus non-TNBC

**Fig. 1:**
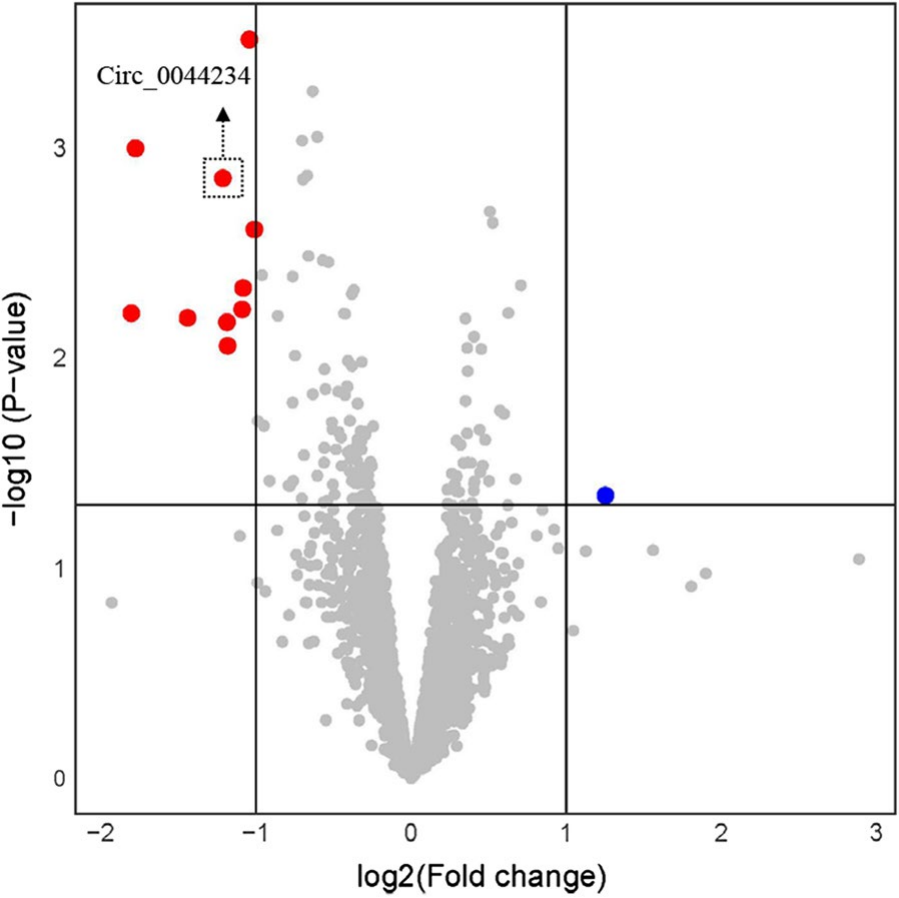
Volcano plot; Differentially expressed circRNAs (DECs) in triple negative breast cancer in comparison with luminal samples from GEO database GSE101124. The vertical and horizontal dashed lines indicate the fold shift (log_2_ scale) and P.value (log_10_ scale) cut-off points, respectively. Red represents downregulated circRNAs and blue represents upregulated circRNAs

Examining the rate of circRNA expression in TNBCs relative to luminal tumors revealed eleven significant DECs, regarding to the microarray dataset GSE101124 (Fig. 1 and Additional file 1: Table S1). The details of DECs have been gathered in Additional file 1: Table S2. The circ_0044234 (hsa_circRNA_102101) was chosen for further study based on P.value and logFC variables (logFC = − 1.213; P.value = 0.001).

### Evaluation of Circ_0044234 expression in the different subtypes of breast cancer

**Fig. 2:**
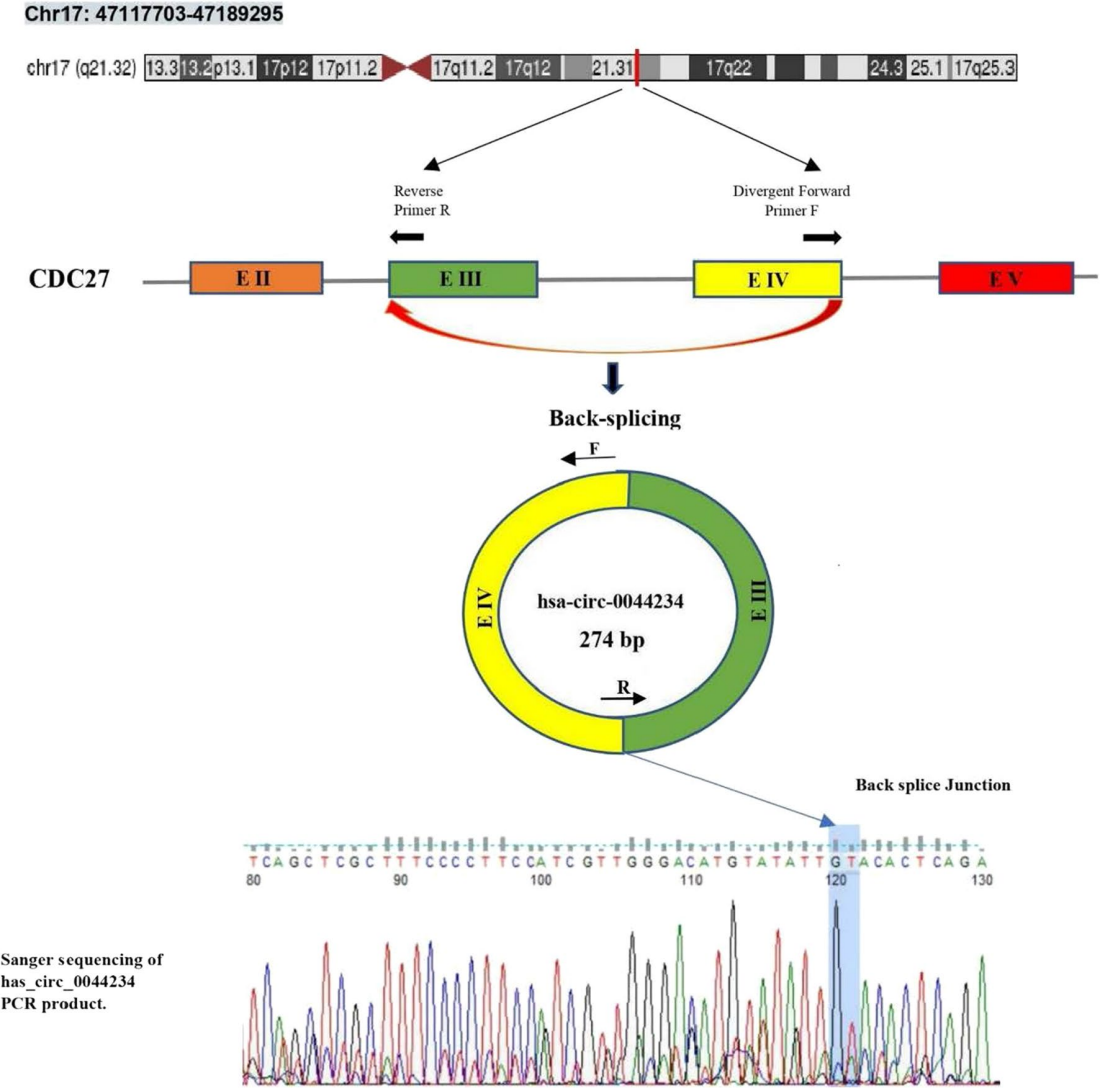
Schematic presentation of hsa_circ_0044234 biogenesis of *CDC27* gene. The verification of back-splice intersection location of hsa_circ_0044234 via Sanger sequencing

**Fig. 3:**
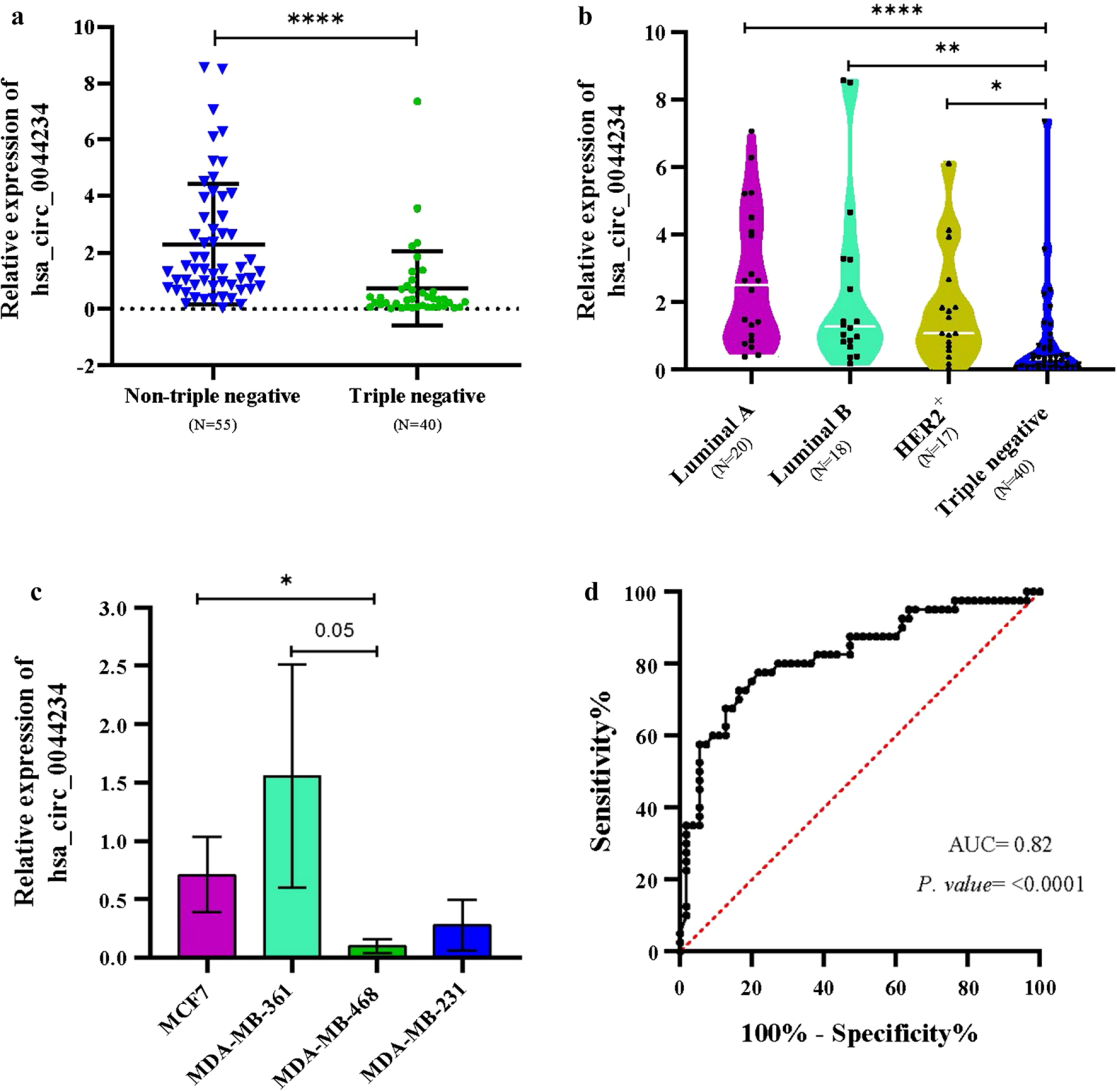
The expression of hsa_circ_0044234 in BC relative to normal mammary tissues. **a** The relative expression levels of circ_0044234 in TNBC in comparison with non-TNBC; **b** the relative expression levels of hsa_circ_0044234 among luminal A/B, HER2^+^ and triple negative breast cancer subtypes; **c** the relative expression levels of hsa_circ_0044234 in triple-negative breast cancer cell lines (MDA-MB-468 and MDA-MB-231) compared to luminal ones (MDA-MB-361 and MCF7); **d** ROC curve analysis of hsa_circ_0044234 expression value for distinguishing TNBC (N = 40) from non-TNBC samples (N = 55); *AUC* area under the ROC curve

We concentrated on circ_0044234, one of the most significant down-expressed DECs in TNBC based on the microarray data, and used quantitative real-time PCR to confirm its expression levels in various subtypes of BC. The back-splicing event from exons 3 to 4 of the *CDC27* gene lead to this exonic circRNA formation which was confirmed by Sanger sequencing (Fig. 2). When TNBC are compared to non-TNBC samples, the expression of circ_0044234 is significantly lower in the former group (Fig. 3a). Moreover, the evaluation of circ_0044234 expressions among four different breast cancer subtypes also reveals a significant down expression of circ_0044234 in triple negative tumors compared to other subtypes (Fig. 3b).

### Evaluation of Circ_0044234 expression in different cell lines of breast cancer

Likewise, we also compared the circ_0044234 expressions in four breast cancer cell lines by quantitative real-time PCR. Results show that MDA-MB-468 significantly expresses the circ_0044234 at a lower level in comparison to MCF7 and MDA-MB-361 cell lines (Fig. 3c). It should be noticed that the MDA-MB-231 cell line expresses circ_0044234 substantially lower than two luminal cell lines. However, these differences have not reached meaningful levels. Overall, the evidence derived from expression analysis in the BC cell lines, in accordance with the tumor samples, supports the remarkable down expression of circ_0044234 as a distinct molecular signature in TNBC.

### Clinical usefulness of Circ_0044234 expression evaluation as a diagnostic and/or prognostic biomarker

The possible clinical usefulness of circ_0044234 in breast cancer patients as a distinctive biomarker of triple negative subtype were assessed by ROC curve analysis. This evaluation reveals that circ_0044234 expressions can be considered as a discriminative biomarker for TNBC subtype (AUC = 0.82; P.value ≤ 0.0001) with 72.5% sensitivity and 83.64% specificity in the optimal cutoff value < 0.64 (Fig. 3d). Moreover, in clinicopathological analyses of the patients, the univariate analyses revealed that lower levels of the circ_0044234 expressions in breast tumors are associated with positive LNM, high Ki67 expression, and higher histological grade (Table 2). Multivariate analysis indicated that there are significant differences (Wilks Lambda P.value = 0.0001, F(4, 71) = 15.26, Value = 0.53) between the two groups of circ_0044234 expression level (low, N = 47 and high, N = 41) at least in terms of one of the dependent variables (histological grade, LMN, Ki67 status, and tumor subtype). According to the results, the two groups of patients with the high or low level of circ_0044234 expression were significantly different from each other in terms of grade (P < 0.001), Ki67 status (P = 0.005), and tumor subtype (P < 0.001) (Additional file 1: Table S3).

**Table 2:** The Association between relative expression of circ_0044234 in breast cancer and clinicopathological features of the patients

Characteristic	Subgroups	No. of patients (%)	Median (expression of circ….)	*P-value*
Age	< 50	54 (56.8%)	1.58	0.717
	≥ 50	41 (43.2%)	1.73	
Tumor size	< 2	18 (19%)	1.88	0.845
	2–5	72 (75.8%)	1.59	
	> 5	5 (5.2%)	1.49	
Estrogen receptor	Positive	38 (40%)	2.54	**0.0002**
	Negative	57 (60%)	1.04	
progesterone receptor	Positive	37 (38.9%)	2.60	**0.0001**
	Negative	58 (61.1%)	1.03	
HER2	Positive	35 (36.8%)	2.03	0.142
	Negative	60 (63.2%)	1.41	
Ki67^a^	High	47 (57.3)	0.62	**< 0.0001**
	Low	35 (42.7)	1.74	
Histologic grade^b^	G1	20 (21.5)	2.52	**0.001**
	G2	47 (50.5)	1.92	
	G3	26 (28)	0.48	
Lymph nodes metastasis^c^	Yes	36 (40)	1.07	**0.030**
	No	54 (60)	2.01	
Histologic type of invasive carcinoma	IDC	68 (94.5%)	0.83	0.140
	ILC	4 (5.5%)	3.22	

*IDC* invasive ductal carcinoma, *ILC* invasive lobular carcinoma, Bold values denote statistical significance at the p < 0.05 level

^a^Data on thirteen patients are lacking. A cut-off point of 25% of positive cells per field was used to distinguish between the categories of high (> 25%) and low (≤ 25%) Ki67 expression

^b^Data on five patients are lacking

^c^Data on two patients are lacking

**Fig. 4:**
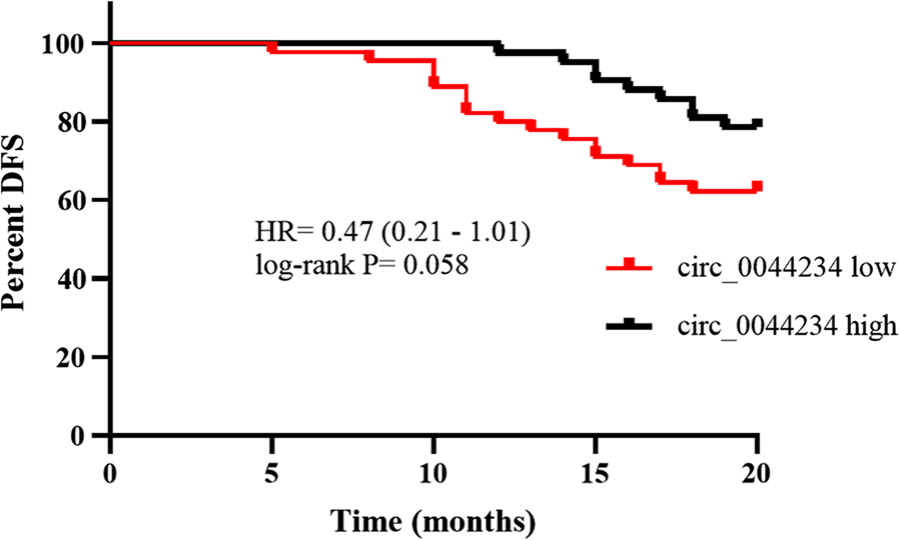
Association of circ_0044234 expression status with disease-free survival in breast cancer. The black and red lines indicate patients with the high (N = 42) and low (N = 45) expression level of circ_0044234, respectively

The Kaplan–Meier method indicates that patients with low circ_0044234 expression have a shorter DFS (Fig. 4). However, the P.value is on the brink of significance (P.value = 0.058).

### The Circ_0044234 as a potential regulator of GATA3 through sponging miR-135b-5p

**Fig. 5:**
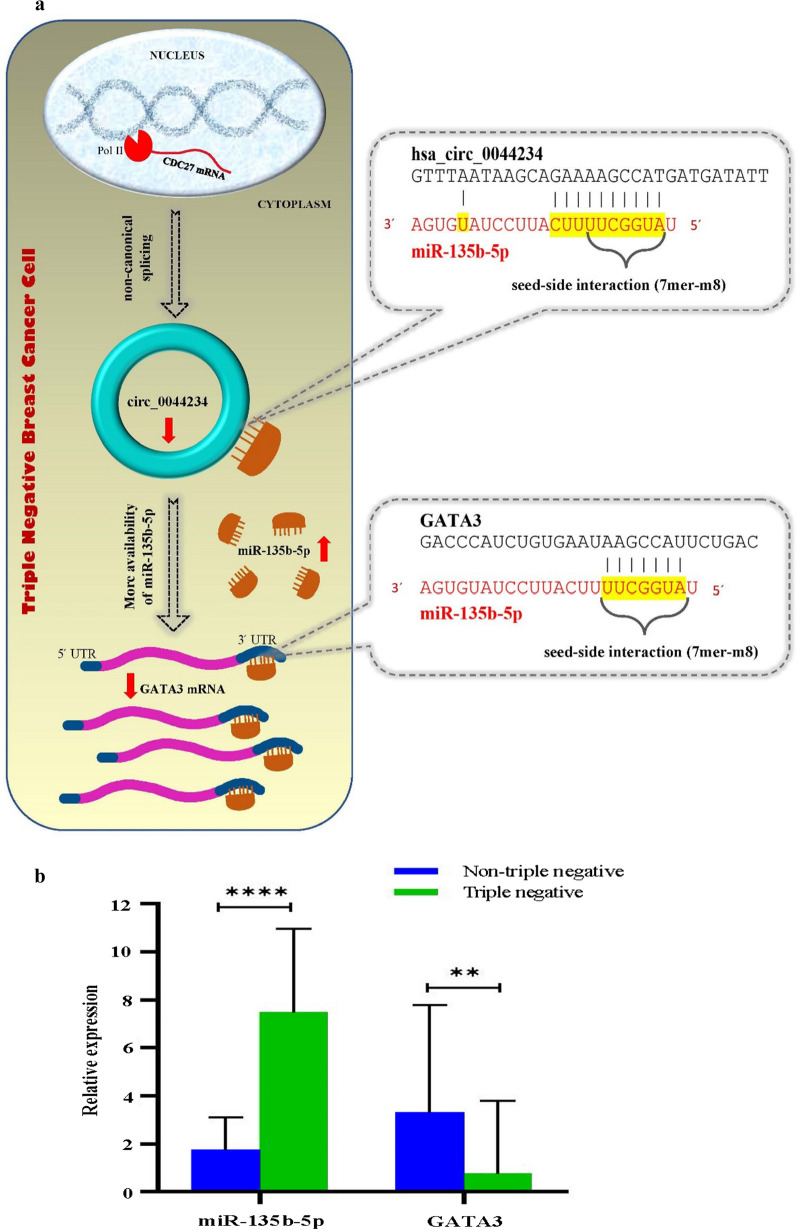
**a** Schematic diagram shows the potential function of circ_0044234 in sponging miR-135b-5p that promotes GATA3 gene; **b** The expression levels of miR-135b-5p and GATA3 in TNBC (N = 40) and non-TNBC (N = 55) relative to normal mammary tissues (N = 10)

**Fig. 6:**
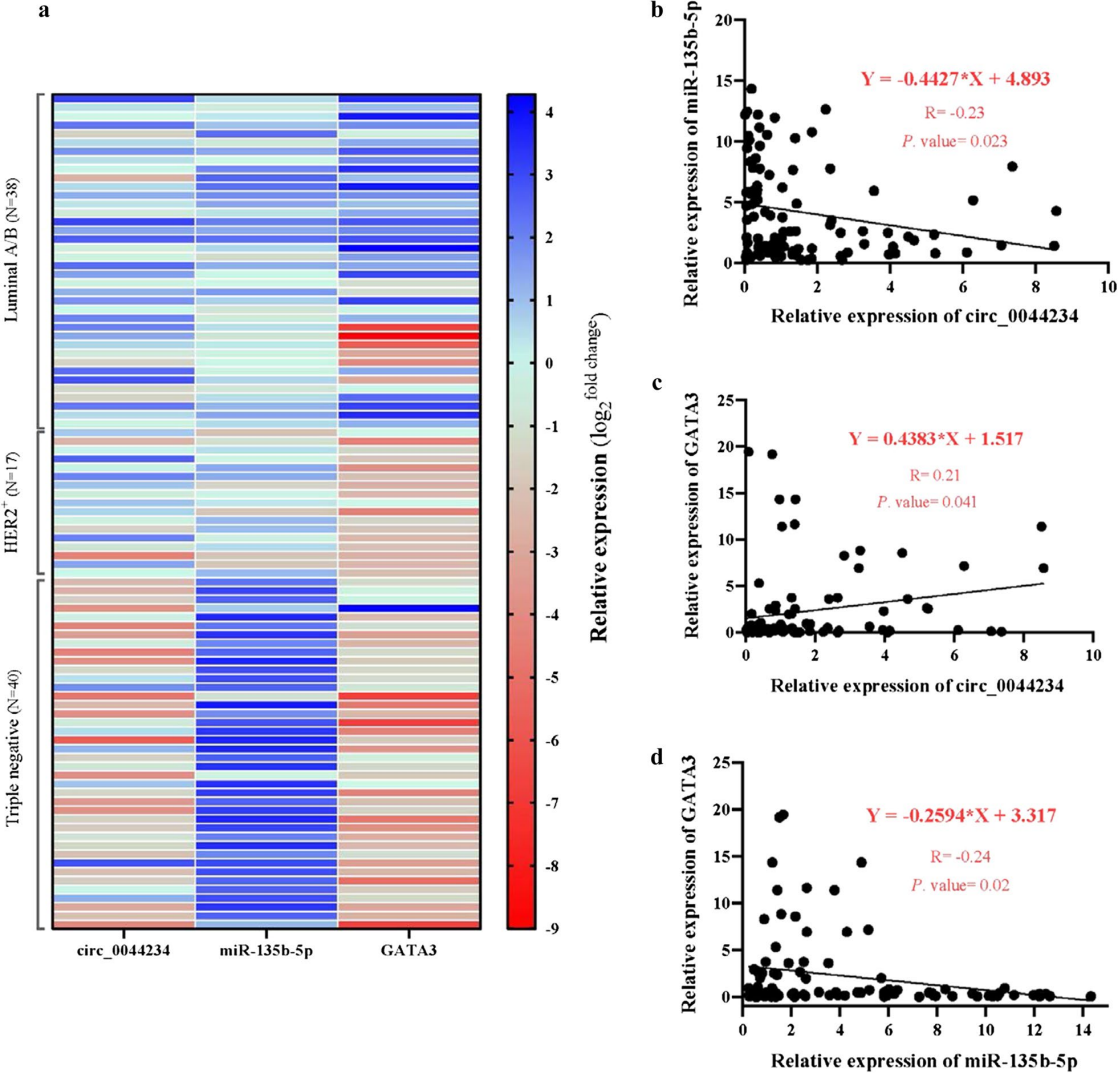
**a** Hierarchical clustering showing expression values (log_2_ transformed) of circ_0044234, miR-135b-5p and GATA3 in different subtype of breast cancers (N = 95) relative to normal mammary tissues (N = 10). Red strips represent low relative expression and blue strips represent high relative expression. Each row shows one tumor sample, and each column demonstrates a transcript; **b** the negative correlation between circ_0044234 and miR-135b-5p expression levels in BC (N = 95, r = − 0.23, P.value = 0.023); **c** The positive correlation between circ_0044234 and GATA3 expression levels in BC (N = 95, r = 0.21, P.value = 0.041); **d** The negative correlation between miR-135b-5p and GATA3 expression levels in BC (N = 95, r = − 0.24, P.value = 0.02); Linear regression analysis was used to achieve the regression equation

We discovered that circ_0044234 harbors a potential MRE for miR-135b-5p (Fig. 5a). According to our previous investigation of the two microarray datasets, GSE19536 and GSE86948, miR-135b-5p is the most up-expressed miRNA in TNBC compared with non-TNBC [16]. Firstly, the up-expression of miR-135b-5p in TNBC was confirmed by quantitative real-time PCR (Fig. 5b). The findings indicate that the upregulation of miR-135b-5p is a remarkable molecular alteration in TNBC. After that, to decide whether circ_0044234 may serve as a sponge for miR-135b-5p in BC, we evaluated the expression correlation between circ_0044234 and miR-135b-5p in the breast tumors (Fig. 6b). There is a negative correlation between expression of these two ncRNAs in the breast tumors (r = -0.23, P.value = 0.023). Regarding the linear regression model, per one-unit decrease in the circ_0044234 expressions could lead to a 4.89-unit increase in the miR-135b-5p expression in BC.

We have bioinformatically predicted that GATA3 is a potential downstream target of circ_0044234/ miR-135b-5p axis and have investigated their expression correlation in the BC samples. The observed positive expression correlation between circ_0044234 and GATA3 as well as the negative expression correlation between miR-135b-5p and GATA3 strengthen this hypothesis (Fig. 6c, d). The level of the GATA3 expression is expected to fall by 3.31 unit for every additional unit in the miR-135b-5p expression in BC (r = − 0.24, P.value = 0.02). Interestingly, as it is shown in Fig. 6a, the heat-map derived from expression data of circ_0044234 /miR-135b-5p/ GATA3 axis reveals a distinctive expression profile between TNBC samples and non-TNBC ones (HER2^+^ and Luminal A/B subtypes).

## Discussion

Bioinformatics studies and high-throughput sequencing are currently detecting a growing number of functional circRNAs [19]. Because of their unique characteristics and distinct advantages, such as their stability, long lifespan, time- and tissue-specific expression, they have turned into a novel hotspot in ncRNAs' research field to serve as potential biomarkers of cancer diagnosis and prognosis. CircRNAs are implicated in the immune response against tumors [20], amplification [21], invasion [22], and critical signaling pathways in BC [23, 24], according to several reports. However, the function of circRNAs in BC cancer cells, is still largely unknown, and more investigations are required. TNBC is recognized as a subtype of BC with high rates of metastasis rate and poor prognosis. Furthermore, relative to other subtypes, treatment strategies for these patients are limited, and disease management remains a challenge [25]. As a result, the implementation of rapid and innovative strategies to increase the diagnostic performance of TNBC is essential. A new microarray analysis found that TNBC had eight times more dysregulation of circRNAs than luminal tumors as compared to normal mammary tissues (162 DECs vs. 20 DECs) [26].

In the current study, from analysis of the GEO dataset, eleven DECs in TNBC in comparison with non-TNBC samples have been detected. Ten out of eleven DECs are down-expressed circRNAs, and just one has an up-expressed manner (Fig. 1). This observation implies that DECs in TNBC mainly act as tumor suppressor ncRNAs. We picked out circ_0044234, one of the most significant down-expressed DECs in TNBC, for a more thorough examination. The circ_0044234 (hsa_circRNA_102101) is a circular transcript of *CDC27* gene with 274 bp length. The changes in CDC27 level have been reported in differing neoplasms. It has been proposed to function as either a tumor suppressor gene or an oncogene [27]. In TNBC, it was reported that CDC27 downregulation is associated with modifying cell proliferation, migration and radio-sensitivity of the cancer cells [28]. However, the functions of circRNAs derived from *CDC27* gene in BC remain largely unknown. Our experimental investigation of circ_0044234 expressions in various BC subtypes as well as cell lines reveals that TNBC expresses circ_0044234 at a substantially lower level than non-TNBC. The ROC curve analysis indicates that it could be applied as a discriminative biomarker to identify TNBC from other BC subtypes. In addition, we examined the prognostic value of circ_0044234 expression in terms of DFS in breast cancer patients. This data reveals that patients with a low expression level of circ_0044234 show a tendency to lower DFS (P.value = 0.058). As the follow-up time in the current study is short (20 months), it is expected that this difference will be likely significant in a larger sample size and a longer follow-up time that needs further research. More importantly, the lower expression of circ_0044234 was positively associated with the higher tumor grade, high Ki67 expression, and positive LNM in BC patients. The multivariate analysis indicates that patients with low and high levels of circ_0044234 are significantly different in terms of histological grade, Ki67 status, and tumor subtype. These findings emphasize that circ_0044234 expression level could serve as an independent prognostic biomarker in BC. Downregulation of circ_0044234 has also been previously highlighted in lupus nephritis (LN) [29, 30], pulmonary and active tuberculosis (TB) patients [29, 31, 32]. Inducing compensatory expression of tumor suppressor circRNAs that undergo a tremendous down expression in tumors might be a novel practical therapeutic strategy [33]. These stable and lightweight molecules can be delivered by nanoparticles [34] or exosomes [35]. Although, at the present time, there are not any clinical reports concerning their effectiveness on cancer-targeted therapies.

Recently, it has been demonstrated that circRNAs can take part in ceRNA networks by harboring MREs and sponging miRNAs [36]. In our previous study, we developed a dysregulated miRNA-mRNA network in TNBC [16]. Based on that research, we screened possible interactions between circ_0044234 and upregulated miRNAs in TNBC. Surprisingly, the circ_0044234 harbors an MRE for miR-135b-5p, the most up-expressed miRNA in TNBC compared with non-TNBC (Fig. 5a). Moreover, we previously demonstrated that GATA3 is potentially a target for miR-135b-5p. The conserved 3́-UTR sequence of GATA3 for miR-135b-5p is demonstrated in Fig. 5a. The miR-135b-5p/GATA3 axis has been illustrated as one of the most eminent interactions in the gene–TF–miRNA regulatory network in TNBC [16]. Here we evaluated their expression correlation with circ_0044234 in BC samples. Our findings suggest a novel potential molecular interaction in the circ_0044234/miR-135b-5p/GATA3 axis as a ceRNA regulatory network in BC (Fig. 5a). Previous studies have demonstrated that miR-135b-5p plays an oncogenic role in several cancers. Bertoli et al. reported that miR-135b-5p has a high degree of centrality in TNBC, and its expression level correlated with tumor cell proliferation, invasion, and migration index [37]. Interestingly, the silencing of the miR-135b-5p in the MDA-MB-231 cell line can decrease cellular mobility and increase the mesenchymal-to-epithelial transition (MET) differentiation process [37]. We suggested that decreases in intracellular levels of circ_0044234 and the subsequent more availability of miR-135b-5p could induce an epithelial-to-mesenchymal transition (EMT) in TNBC cells. In future studies, this hypothesis could be evaluated in MCF10-A through the treatment with TGF-beta or with other factors that induce an EMT/MET program [38].

Paolo Uva et al. suggested that miR-135b-5p may be a therapeutic target in basal-like TNBC [39].

GATA3 is a transcription factor that regulates the gene expression involved in the differentiation of mammary epithelial cells, and progression and metastasis in BC [40]. *GATA3* is one of the most down-regulated genes in TNBC. However, the precise mechanisms underlying the dysregulation of this gene in TNBC have remained chiefly elusive. The down expression of GATA3 in BC is linked to a tumor phenotype that is prone to invasive growth and has a poor prognosis [41]. It is proposed that GATA3 is required for homologous recombination DNA repair, which is one of the most disrupted pathways in TNBC [42, 43].

Collectively, the bioinformatics investigation, besides experimental evidence, raises an exciting hypothesis that circ_0044234 could regulate GATA3 via serving as a sponge of miR-135b-5p in BC. Our expression correlation study suggests for the first time that GATA3 dysregulation can be controlled as a key downstream target of the circ_0044234/miR-135b-5p axis in BC. As GATA3 is a crucial transcription factor in BC, the lack of its expression could fundamentally affect the onco-transcriptomic profile of triple negative tumors. Hence, the circ_0044234/miR-135b-5p axis might be a promising therapeutic target for TNBC patients.

## Conclusion

For the first time, we have identified circ_0044234 as a noticeable down-expressed circRNA in TNBC compared with other BC subtypes and could be a promising diagnostic and prognostic biomarker. In addition, the novel circ_0044234/miR-135b-5p/GATA3 axis in BC is proposed through our bioinformatics and experimental approach which provides worthwhile insight into the potential mechanism of circ_0044234-mediated gene silencing in TNBC. Further experimental validations are required to elucidate the functional roles of circ_0044234 in TNBC.

## Supplementary Information

**Additional file 1: Table S1.** Analysis of DECs in TNBT versus Luminal Tumors based on microarray dataset GSE101124. **Table S2.** Characteristic of differentially-expressed circRNAs (DECs) in triple negative breast tumor (TNBT) compared with non-TNBT samples. **Table S3.** MANOVA test for comparing the two groups of high and low levels of circ_0044234 expression in terms of the tumor grade, lymph nodes metastasis (LNM), Ki67 expression status, and tumor subtype (TNBC and nonTNBC).

## Data Availability

The datasets supporting the conclusions of this article are available in: [GEO dataset] at (https://www.ncbi.nlm.nih.gov/geo/). The datasets supporting the conclusions of this article are also included within the article and its additional files.

## Abbreviations

BC: Breast cancer
circRNA: Circular RNA
ncRNA: Non-coding RNA
miRNA: MicroRNA
ceRNA: Competing endogenous RNA
TNBC: Triple negative breast cancer
HER2: Human epidermal growth factor receptor 2
ER: Estrogen receptor
PR: Progesterone receptor
MRE: MiRNA response element
DEC: Differentially expressed circRNA
DFS: Disease-free survival
LNM: Lymph node metastasis
MANOVA: Multivariate analysis of variance
EMT: Epithelial-to-mesenchymal transition
MET: Mesenchymal-to-epithelial transition

## References

[1] Sung H, Ferlay J, Siegel RL, Laversanne M, Soerjomataram I, Jemal A (2021). Global cancer statistics 2020: GLOBOCAN estimates of incidence and mortality worldwide for 36 cancers in 185 countries. CA: Cancer J Clin..

[2] Prat A, Pineda E, Adamo B, Galván P, Fernández A, Gaba L (2015). Clinical implications of the intrinsic molecular subtypes of breast cancer. Breast J.

[3] Sims AH, Howell A, Howell SJ, Clarke RB (2007). Origins of breast cancer subtypes and therapeutic implications. Nat Rev Clin Oncol.

[4] Foulkes WD, Smith IE, Reis-Filho JS (2010). Triple-negative breast cancer. N Engl J Med.

[5] Lin NU, Claus E, Sohl J, Razzak AR, Arnaout A, Winer EP (2008). Sites of distant recurrence and clinical outcomes in patients with metastatic triple-negative breast cancer: high incidence of central nervous system metastases. Cancer.

[6] Jalalvand M, Darbeheshti F, Rezaei N (2021). Immune checkpoint inhibitors: review of the existing evidence and challenges in breast cancer. Immunotherapy.

[7] Collignon J, Lousberg L, Schroeder H, Jerusalem G (2016). Triple-negative breast cancer: treatment challenges and solutions. Breast Cancer Targets Ther.

[8] Elwan A, Abdelrahman AE, Alnagar AA, Abdelhamid MI, Nawar N (2021). Clinicopathological features and treatment challenges in triple negative breast cancer patients: a retrospective cohort study. Turk J Ophthalmol.

[9] Kouhsar M, Jamalkandi SA, Moeini A, Masoudi-Nejad A (2019). Detection of novel biomarkers for early detection of Non-Muscle-Invasive Bladder Cancer using Competing Endogenous RNA network analysis. Sci Rep.

[10] Xiao MS, Ai Y, Wilusz JE (2020). Wilusz, biogenesis and functions of circular RNAs come into focus. Trends Cell Biol.

[11] Quan G, Li J (2018). Circular RNAs: biogenesis, expression and their potential roles in reproduction. J Ovarian Res.

[12] Kulcheski FR, Christoff AP, Margis R (2016). Circular RNAs are miRNA sponges and can be used as a new class of biomarker. J Biotechnol.

[13] Jahani S, Nazeri E, Majidzadeh-A K, Jahani M, Esmaeili R (2020). Circular RNA; a new biomarker for breast cancer: a systematic review. J Cell Physiol.

[14] Lü L, Sun J, Shi P, Kong W, Xu K, He B (2017). Identification of circular RNAs as a promising new class of diagnostic biomarkers for human breast cancer. Oncotarget.

[15] Nair AA, Niu N, Tang X, Thompson KJ, Wang L, Kocher JP (2016). Circular RNAs and their associations with breast cancer subtypes. Oncotarget.

[16] Darbeheshti F, Rezaei N, Amoli MM, Mansoori Y, Tavakkoly BJ (2019). Integrative analyses of triple negative dysregulated transcripts compared with non-triple negative tumors and their functional and molecular interactions. J Cell Physiol.

[17] Xia S, Feng J, Chen K, Ma Y, Gong J, Cai F (2018). CSCD: a database for cancer-specific circular RNAs. Nucleic acids Res.

[18] Dudekula DB, Panda AC, Grammatikakis I, De S, Abdelmohsen K, Gorospe M (2016). CircInteractome: a web tool for exploring circular RNAs and their interacting proteins and microRNAs. RNA Biol.

[19] Kadkhoda S, Darbeheshti F, Tavakkoly-Bazzaz J (2020). Identification of dysregulated miRNAs-genes network in ovarian cancer: An integrative approach to uncover the molecular interactions and oncomechanisms. Cancer Rep.

[20] Paramasivam A, Priyadharsini JV (2020). Novel insights into m6A modification in circular RNA and implications for immunity. Cell Mol Immunol.

[21] Wang L, Yi J, Lu LY, Zhang YY, Wang L, Hu GS (2021). Estrogen-induced circRNA, circPGR, functions as a ceRNA to promote estrogen receptor-positive breast cancer cell growth by regulating cell cycle-related genes. Theranostics.

[22] Chen L, Nan A, Zhang N, Jia Y, Li X, Ling Y (2019). Circular RNA 100146 functions as an oncogene through direct binding to miR-361-3p and miR-615-5p in non-small cell lung cancer. Mol Cancer.

[23] Zhang XY, Mao L (2021). Circular RNA Circ_0000442 acts as a sponge of MiR-148b-3p to suppress breast cancer via PTEN/PI3K/Akt signaling pathway. Gene.

[24] Wang X, Ji C, Hu J, Deng X, Zheng W, Yu Y (2021). Hsa_circ_0005273 facilitates breast cancer tumorigenesis by regulating YAP1-hippo signaling pathway. J Exp Clin Cancer Res.

[25] Dass SA, Tan KL, Selva Rajan R, Mokhtar NF, Mohd Adzmi ER, Wan Abdul Rahman WF (2021). Triple negative breast cancer: a review of present and future diagnostic modalities. Medicina.

[26] Xu JZ, Shao CC, Wang XJ, Zhao X, Chen JQ, Ouyang YX (2019). circTADA2As suppress breast cancer progression and metastasis via targeting miR-203a-3p/SOCS3 axis. Cell death Dis.

[27] Kazemi-Sefat GE, Keramatipour M, Talebi S, Kavousi K, Sajed R, Kazemi-Sefat NA (2021). The importance of CDC27 in cancer: molecular pathology and clinical aspects. Cancer Cell Int.

[28] Ren YQ, Fu F, Han J (2015). MiR-27a modulates radiosensitivity of triple-negative breast cancer (TNBC) cells by targeting CDC27. Med Sci Monit.

[29] Huang ZK, Yao FY, Xu JQ, Deng Z, Su RG, Peng YP (2018). Microarray expression profile of circular RNAs in peripheral blood mononuclear cells from active tuberculosis patients. Cell Physiol Biochem.

[30] Ouyang Q, Huang Q, Jiang Z, Zhao J, Shi GP, Yang M (2018). Using plasma circRNA_002453 as a novel biomarker in the diagnosis of lupus nephritis. Mol Immunol.

[31] Liu H, Lu G, Wang W, Jiang X, Gu S, Wang J (2020). A panel of circRNAs in the serum serves as biomarkers for mycobacterium tuberculosis infection. Front Microbiol.

[32] Ojha R, Nandani R, Chatterjee N, Prajapati VK (2018). Emerging role of circular RNAs as potential biomarkers for the diagnosis of human diseases. Circ RNAs.

[33] Zhou R, Wu Y, Wang W, Su W, Liu Y, Wang Y (2018). Circular RNAs (circRNAs) in cancer. Cancer Lett.

[34] Du WW, Fang L, Yang W, Wu N, Awan FM, Yang Z (2017). Induction of tumor apoptosis through a circular RNA enhancing Foxo3 activity. Cell Death Differ.

[35] Zhang M, Xin Y (2018). Circular RNAs: a new frontier for cancer diagnosis and therapy. J Hematol Oncol.

[36] Kadkhoda S, Darbeheshti F, Rezaei N, Azizi-Tabesh G, Zolfaghari F, Tavakolibazaz S (2021). Investigation of circRNA-miRNA-mRNA network in colorectal cancer using an integrative bioinformatics approach. Gastroenterol Hepatol Bed Bench.

[37] Bertoli G, Cava C, Corsi F, Piccotti F, Martelli C, Ottobrini L (2021). Triple negative aggressive phenotype controlled by miR-135b and miR-365: new theranostics candidates. Sci Rep.

[38] Pattabiraman DR, Bierie B, Kober KI, Thiru P, Krall JA, Zill C (2016). Activation of PKA leads to mesenchymal-to-epithelial transition and loss of tumor-initiating ability. Science.

[39] Uva P, Cossu-Rocca P, Loi F, Pira G, Murgia L, Orrù S (2018). miRNA-135b contributes to triple negative breast cancer molecular heterogeneity: different expression profile in Basal-like versus non-Basal-like phenotypes. Int J Med Med Sci.

[40] Shahi P, Wang CY, Chou J, Hagerling C, Velozo HG, Ruderisch A (2017). GATA3 targets semaphorin 3B in mammary epithelial cells to suppress breast cancer progression and metastasis. Oncogene.

[41] Afzaljavan F, Sadr AS, Savas S, Pasdar A (2021). GATA3 somatic mutations are associated with clinicopathological features and expression profile in TCGA breast cancer patients. Sci Rep.

[42] Darbeheshti F, Izadi P, Razavi AN, Yekaninejad MS, Bazzaz JT (2018). Comparison of BRCA1 expression between triple-negative and luminal breast tumors. Iran Biomed J.

[43] Zhang F, Tang H, Jiang Y, Mao Z (2017). The transcription factor GATA3 is required for homologous recombination repair by regulating CtIP expression. Oncogene.

